# Two-year follow-up of patients with myocardial injury during acute COVID-19: insights from the CARDIO COVID 20–21 registry

**DOI:** 10.3389/fcvm.2025.1584732

**Published:** 2025-06-11

**Authors:** Juan Pablo Arango-Ibanez, Brayan Daniel Cordoba-Melo, Mario Miguel Barbosa Rengifo, Jesika Daniela Tobar-Arteaga, Maria Lucia Castro-Trujillo, Cesar José Herrera, Miguel Ángel Quintana Da Silva, Andrés Felipe Buitrago Sandoval, María Lorena Coronel Gilio, Freddy Pow Chon Long, Liliana Cárdenas Aldaz, Andrea Valencia, Carlos Enrique Vesga-Reyes, Juan Esteban Gómez-Mesa

**Affiliations:** ^1^ Centro de Investigaciones Clínicas, Fundación Valle del Lili, Cali, Valle del Cauca, Colombia; ^2^ Facultad de Ciencias de la Salud, Universidad Icesi, Cali, Valle del Cauca, Colombia; ^3^ Departamento de Cardiología, Centros de Diagnóstico y Medicina Avanzada y de Conferencias Médicas y Telemedicina (CEDIMAT), Santo Domingo, República Dominicana; ^4^ Departamento de Cardiología, Instituto Cardiovascular Sanatorio MIGONE, Asunción, Paraguay; ^5^ Departamento de Cardiología, Fundación Santa Fe, Bogotá, Colombia; ^6^ Departamento de Cardiología, Instituto de Cardiología J. F. Cabral, Corrientes, Argentina; ^7^ Departamento de Cardiología, Hospital Luis Vernaza, Guayaquil, Ecuador; ^8^ Departamento de Cardiología, Hospital Eugenio Espejo, Quito, Ecuador; ^9^ Departamento de Cardiología, Fundación Valle del Lili, Cali, Valle del Cauca, Colombia

**Keywords:** COVID-19, myocardial injury, complications, prognosis, survivors

## Abstract

**Introduction:**

COVID-19 can cause Myocardial Injury (MI) during acute illness, which has been strongly associated with worse outcomes during hospitalization, however, more research is required on its effects on long-term outcomes, especially in underexplored regions in the literature such as Latin America.

**Methods:**

This multicenter prospective cohort study follows up with patients with previous severe COVID-19 at a 2-year follow-up encounter. Comprehensive assessments were conducted including demographic data, clinical variables, psychiatric evaluations, and echocardiographic studies. Patients were stratified by the presence or absence of MI during their acute COVID-19 hospitalization. Statistical analyses included logistic regression and univariate comparisons.

**Results:**

Of the 210 patients included, 53 (25%) had MI. Patients with MI were older, had a higher prevalence of comorbidities (e.g., hypertension, chronic kidney disease, atrial fibrillation), and were more likely to require intensive care unit admission, invasive mechanical ventilation, and vasopressor or inotropic support during acute COVID-19. Regarding long-term cardiovascular outcomes, no significant differences were observed in *de novo* cardiovascular disease, venous thromboembolism, or acute cardiovascular events. Patients with MI had greater odds of cardiopulmonary hospitalizations during follow-up (aOR 3.67, 95% CI 1.07–13.07, *p* = 0.037) after adjusting for age and sex.

**Conclusion:**

Patients with prior MI during COVID-19 had a higher prevalence of comorbidities, poorer functional status, and increased odds of cardiopulmonary hospitalizations over a two-year follow-up evaluation compared to those without MI. Although prior studies suggest an association between MI and worse long-term outcomes, the evidence remains inconsistent. These findings emphasize the need for ongoing research to clarify how MI contributes to worsened long-term outcomes.

## 1 Introduction

By 2024, the COVID-19 pandemic had resulted in over 7 million deaths worldwide (1). COVID-19 primarily causes upper respiratory infections in mild cases, but moderate and severe cases can progress to pneumonia and multiorgan failure. While the respiratory system is the primary target of SARS-CoV-2, the virus can significantly affect various other organs (2). Strong evidence underscores the profound impact of COVID-19 on the cardiovascular system, driven by cytokine release, hypoxia, coagulopathy, endothelial damage, and the exacerbation of pre-existing cardiovascular conditions (3, 4). These can lead to cardiovascular complications such as arrhythmias, acute heart failure (AHF), pulmonary embolism (PE), an myocardial injury (MI), among many others (5, 6).

MI caused by COVID-19 is defined as detecting cardiac troponin levels exceeding the 99th percentile of the upper reference range. This condition is caused by direct viral invasion, inflammation, endothelial damage, and microvascular thrombosis (6). The incidence of acute MI in hospitalized COVID-19 patients ranges from 16.1%–23.8% (7) and it is associated with worse clinical outcomes including higher rates of intensive care unit (ICU) admission and in-hospital mortality (8–12). Factors associated with MI include older age, male sex, and having pre-existing comorbidities (11). Studies demonstrate that COVID-19 patients with MI have worse clinical outcomes in the long term, for instance, an increased risk of cardiovascular complications, hospital readmissions, and death (13, 14).

Having a deeper understanding of the long-term consequences of MI in patients is crucial given the rising cardiovascular burden caused by long COVID (15). Furthermore, assessing the chronic impact of COVID-19 in heavily affected regions, such as Latin America, is essential, particularly given the limited research output from this area (16, 17). To help fill this gap, we conducted a prospective analysis of previously hospitalized COVID-19 patients from four Latin American countries, comparing those who experienced MI during the acute event to those who did not. We aim for this study to inform public health policies for effective risk stratification and surveillance of patients at higher risk of long-term consequences of COVID-19.

## 2 Materials and methods

### 2.1 Study design and participants

A prospective cohort study was designed using data from the CARDIO COVID 19–20 registry (18), which is a multicenter database comprising 3,260 hospitalized patients with microbiologically confirmed COVID-19 from 44 institutions across 14 countries. Patients in that study were enrolled between June 1, 2020, and June 1, 2021. Institutions involved in this registry were invited to participate in a subsequent follow-up study, CARDIO COVID 20–21, to evaluate long-term outcomes, including persistent symptoms, and radiological abnormalities. The coordination and oversight of both registries were undertaken by the Inter-American Council of Heart Failure and Pulmonary Hypertension (CIFACAH) under the guidance of the Inter-American Society of Cardiology (IASC).

For this subanalysis, we included patients with previous severe COVID-19 during hospitalization who signed informed consent to participate. We excluded patients without baseline troponin results. Participants of this study were evaluated between September 2022 and February 2023.

### 2.2 Definitions

Severe COVID-19 was defined as the presence of one or more of the following: the need for ICU admission, MI indicated by elevated troponin levels, high risk of venous thromboembolism (VTE) evidenced by elevated D-dimer levels, or the development of new cardiovascular complications during hospitalization, including AHF, stroke, or PE.

Troponin measurements in the original CARDIO COVID 19–20 registry were performed according to the clinical protocols of the participating institutions. Universal screening was not mandated, leading to testing primarily in patients with higher clinical suspicion of cardiac involvement, greater disease severity (e.g., ICU admission), or specific institutional practices. Acute MI was defined when detecting a high-sensitivity cardiac troponin (hs-cTn) level equal to or greater than the 99th percentile during the acute COVID-19 hospitalization. In our study, the following thresholds were used: for hs-cTn I, 34.2 ng/L in men and 15.6 ng/L in women; for hs-cTn T, 100 ng/L; and for conventional troponin I, 33 ng/L in men and 13 ng/L in women.

Troponin levels were measured at admission, discharge, or both, depending on clinical criteria. Among the 210 patients with troponin data, 136 had measurements only at admission, 4 only at discharge, and 70 at both time points. A troponin elevation at any time during hospitalization was considered positive. The detailed distribution of troponin positivity according to timing of measurement is provided in Supplementary Table S1 of the Supplementary Material.

MI was defined if any of the values exceeded the specified thresholds. In this cohort, 88.8% of patients were evaluated using hs-cTn I, 5.8% with conventional troponin I, and 1.5% with hs-cTn T.

Cardiopulmonary in-patient hospitalization was defined as any admission including a cardiopulmonary complaint (e.g., arrhythmias, acute coronary syndrome, PE) excluding malignant or infectious conditions.

### 2.3 Data collection

For the CARDIO COVID 20–21 registry, institutions involved in the previous CARDIO COVID 19–20 study (44 institutions across 14 countries) were invited to join the extended follow-up phase. Of these, 7 institutions from 5 countries agreed to participate and institutions from 4 of those countries contributed to patients for this study. Patients were initially reached by phone and invited to enroll in the study. Upon agreement, informed consent was obtained, followed by a clinical evaluation conducted either in person or over the phone. Patients were invited to participate in the complete study, which involved collecting clinical data, conducting physical examinations, performing psychiatric assessments, and echocardiographic analysis. However, they could choose not to participate in specific sub-analyses, such as psychiatric assessments, or cardiological imaging.

To assess symptoms, we used a standardized questionnaire for self-reported symptoms. The symptom list included those commonly reported in patients with long COVID by the time the study was designed, along with space to document any additional symptoms. Patients were asked about the presence of specific symptoms they experienced within the three months before the follow-up. We evaluated the presence of comorbidities and clinical events at the follow-up by reviewing clinical records. Physical examination data, such as vital signs, were collected by physicians participating in the study during in-person visits.

Psychiatric evaluations were also conducted by physicians participating in this study. We evaluated the following risk scores: General Anxiety Disorder-7 (GAD-7), Patient Health Questionnaire-9 (PHQ-9), and Perceived Stress Scale-14 (PSS-14). GAD-7 is a validated screening tool used to assess the severity of generalized anxiety symptoms. Scores are categorized as follows: 0–4 indicates minimal or no anxiety, 5–9 indicates mild anxiety, 10–14 indicates moderate anxiety, and scores of 15 or higher indicate severe anxiety. The PHQ-9 is a 9-item scale used to screen for depression severity. Scores are interpreted as follows: 0–4 indicates minimal or no depression, 5–9 mild depression, 10–14 moderate depression, 15–19 moderately severe depression, and scores of 20 or higher indicate severe depression. The PSS-14 is a 14-item questionnaire used to assess perceived stress levels. Scores are categorized as follows: 0–13 indicates low perceived stress, 14–26 moderate perceived stress, and 27–40 high perceived stress.

Transthoracic echocardiograms were performed by cardiologists with dedicated expertise in echocardiography. All studies were conducted using echocardiographic systems manufactured by General Electric Company, including the Vivid E9, Vivid XD Clear, Vivid S70, and Vivid I95 models. Examinations were carried out by multiple trained echocardiographers, following standardized institutional protocols to ensure consistency in image acquisition and interpretation. The procedures adhered to the “Guidelines for Performing a Comprehensive Transthoracic Echocardiographic Examination in Adults: Recommendations from the American Society of Echocardiography” (19). All images were reviewed and analyzed on a dedicated workstation using AGFA PACS version 8.2.2.050.

### 2.4 Statistical analysis

A descriptive analysis was performed, with continuous variables summarized as medians and interquartile ranges (IQR) and categorical variables expressed as frequencies and percentages. Normality was evaluated using the Shapiro–Wilk test. Univariate analyses were conducted using Fisher's Exact Test, Pearson's Chi-squared test, and Wilcoxon rank sum test according to the data type. Odds ratios were estimated using a logistic regression analysis to evaluate differences in the rate of cardiopulmonary hospitalizations, adjusting for age and male sex. A two-sided alpha of 0.05 was set for statistical significance. Data analysis was conducted with RStudio 2024.12.0 + 467 (R Foundation for Statistical Computing, Vienna, Austria). Figures were created with Lucidchart and Python version 3.13.1.

### 2.5 Ethical considerations

The study received approval from the Institutional Review Board, Comité de Ética e Investigación of Fundación Valle del Lili (protocol code 1756). Informed consent was obtained from all participants. The study complies with the principles outlined in the Declaration of Helsinki.

## 3 Results

**Figure 1:**
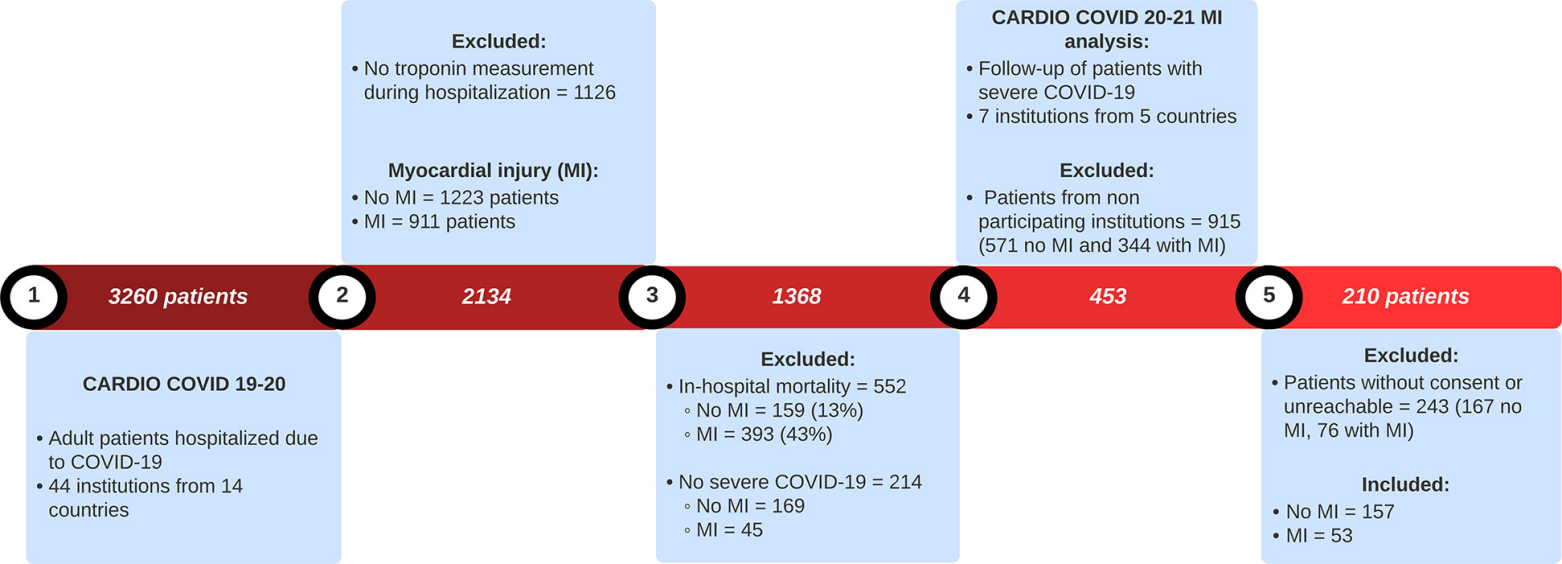
Flowchart of patient selection for CARDIO COVID 20-21 MI analysis.

The CARDIO COVID 19–20 study included 3,260 patients, of whom 1,126 did not have troponin measurements during hospitalization. Among those with troponin measurements, 1,223 showed no evidence of MI, while 911 were diagnosed with MI. In-hospital mortality was recorded in 159 patients without MI and 393 patients with MI. Additionally, 214 patients who did not develop severe COVID-19 during hospitalization were excluded from the analysis (Figure 1).

For the CARDIO COVID 20–21 MI analysis, seven institutions across five countries participated in the study. A total of 915 patients were excluded because they were treated in non-participating institutions. Subsequently, 243 additional patients were excluded due to being unreachable or declining consent, including 167 without MI and 76 with MI. Ultimately, 210 patients were included in the final analysis, comprising 157 without MI and 53 with MI.

We performed a comparison between those who did and did not undergo troponin measurement during acute COVID-19 (Supplementary Table S2 of the Supplementary Material). Patients who had troponin measurements during acute COVID-19 differed significantly from those who did not. They had higher rates of comorbidities, notable differences in baseline medication use and in-hospital management, and were more likely to require ICU admission.

In addition, we evaluated differences between included and excluded patients with available troponin measurement treated at participating institutions (Supplementary Table S3 of the Supplementary Material). Patients excluded did not differ systematically from included patients in terms of demographics, comorbidities, baseline medications, management during acute COVID-19, and ICU admission rates.

To assess the recruitment rate of survivors of severe COVID-19 with troponin measurements, it is important to note that 324 patients without MI and 129 patients with MI were discharged from participating institutions. Among these, 48.4% of patients without MI and 41% of those with MI were included in the follow-up analysis. This reflects a 7.4% lower inclusion rate for the MI group, corresponding to roughly 9 fewer patients.

The recruitment rate by country was as follows: 157 (74.8%) from Colombia, 25 (11.9%) from Paraguay, 22 (10.5%) from the Dominican Republic, 3 (1.4%) from Argentina, and 3 from Ecuador (1.4%). The median follow-up visit of recruited patients was at 25 (IQR, 24–27) months after patient discharge. During this visit, all required evaluations (e.g., echocardiography, psychiatric assessment) were conducted once the patient agreed to participate in them.

### 3.1 Patient demographic and comorbidities

Table 1 demonstrates the demographic characteristics and comorbidities evaluated at a follow-up. The median age of the cohort was 58 (IQR, 49.3, 69), and most of the patients were male (61.9%). A higher prevalence of comorbidities was observed for the group of patients with previous MI. Substantial differences were observed for arterial hypertension (HTN) (*P* = 0.039), sedentarism (*P* = 0.005), chronic kidney disease (CKD) (*P* = 0.017), and atrial fibrillation (AF) (*P* = 0.008).

**Table 1:** Demographics and comorbidities at follow-up stratified by MI.

Variable	Overall, *N* = 210	No MI, *n* = 157	MI, *n* = 53	*P*-value*
Age (years)	58.0 (49.3, 69.0)	57.0 (48.0, 69.0)	61.0 (56.0, 70.0)	0.090
Sex (Male)	130 (61.9%)	100 (63.7%)	30 (56.6%)	0.4
Sedentarism	83 (39.5%)	53 (33.8%)	30 (56.6%)	0.005
Arterial hypertension	115 (54.8%)	79 (50.3%)	36 (67.9%)	0.039
Obesity	114 (54.3%)	83 (52.9%)	31 (58.5%)	0.6
Dyslipidemia	69 (32.9%)	50 (31.8%)	19 (35.8%)	0.7
Diabetes mellitus	64 (30.5%)	44 (28.0%)	20 (37.7%)	0.2
Chronic kidney disease	26 (12.4%)	14 (8.9%)	12 (22.6%)	0.017
Coronary artery disease	17 (8.1%)	9 (5.7%)	8 (15.1%)	0.062
Transplant	15 (7.1%)	11 (7.0%)	4 (7.5%)	>0.9
Heart failure	9 (4.3%)	4 (2.5%)	5 (9.4%)	0.080
Atrial fibrillation	11 (5.2%)	4 (2.5%)	7 (13.2%)	0.008
Smoking (current)	3 (1.4%)	3 (1.9%)	0 (0.0%)	0.573
Smoking (history)	25 (11.9%)	18 (11.5%)	7 (13.2%)	0.807
Stroke	4 (1.9%)	4 (2.5%)	0 (0.0%)	0.6

Values are shown in absolute frequency and percentage, or median and interquartile range.

* Fisher's exact test, Pearson's Chi-squared test; Wilcoxon rank sum test.

### 3.2 Clinical outcomes during acute COVID-19

Table 2 demonstrates the clinical outcomes of the patients during acute COVID-19 admission, stratified by MI groups. Admission to the ICU occurred in 82 (52.2%) patients with no MI and 40 (75.5%) with MI (*P* = 0.005). Patients with MI had higher rates of invasive mechanical ventilation (IMV) and use of inotropic medications (*P* < 0.001). The use of vasopressor medications was more common in patients with MI (50.9% vs. 20.4%) (*P* = 0.063).

**Table 2:** Clinical outcomes during acute COVID-19 admission.

Variable	Overall, *N* = 210	No MI, *n* = 157	MI, *n* = 53	*P*-value*
Admitted to the ICU	122 (58.1%)	82 (52.2%)	40 (75.5%)	0.005
Invasive mechanical ventilation	59 (28.1%)	32 (20.4%)	27 (50.9%)	<0.001
Use of vasopressor	36 (17.1%)	22 (14.0%)	14 (26.4%)	0.063
Use of inotropic	10 (4.8%)	2 (1.3%)	8 (15.1%)	<0.001

ICU, intensive care unit.

* Pearson's Chi-squared test. ICU, intensive care unit.

### 3.3 Symptoms, physical exam, and psychiatric evaluation at long-term follow-up

We found a high prevalence of symptoms in our cohort of patients, with fatigue (56.2%), myalgia/arthralgia (42.9%), and shortness of breath (24.3%) being the most common. No substantial differences were seen when stratifying by MI. Most patients had a New York Heart Association (NYHA) classification I or II (87.7%). Differences were seen in NYHA classifications comparing MI groups (*P* = 0.003). We observed that patients with previous MI had a higher prevalence of a NYHA III (15.1%) and IV (3.8%) classification. The median body-mass index of the cohort was 183 (IQR, 19, 8). We found no differences concerning systolic blood pressure, diastolic blood pressure, pulse rate, and oxygen saturation between MI groups. Patients with MI had a higher prevalence of lower limb edema (31.8% vs. 15.2%) (*P* = 0.03). These findings are described in Table 3. Physical exam variables had missing data (between 12.8% and 19.5%).

**Table 3:** Clinical evaluation at long-term follow-up.

Variable	N for analysis	Overall, *N* = 210	No MI, *n* = 157	MI, *n* = 53	*P*-value*
Symptoms	210				
Fatigue		118 (56.2%)	89 (56.7%)	29 (54.7%)	>0.9
Myalgia/Arthralgia		90 (42.9%)	69 (43.9%)	21 (39.6%)	0.7
Shortness of breath		51 (24.3%)	33 (21.0%)	18 (34.0%)	0.086
Chest pain		46 (21.9%)	33 (21.0%)	13 (24.5%)	0.7
Palpitations		50 (23.8%)	34 (21.7%)	16 (30.2%)	0.3
NYHA	210				
I		132 (62.9%)	108 (68.8%)	24 (45.3%)	0.003
II		52 (24.8%)	33 (21.0%)	19 (35.8%)	
III		24 (11.4%)	16 (10.2%)	8 (15.1%)	
IV		2 (1.0%)	0 (0.0%)	2 (3.8%)	
Physical exam					
Body mass index (BMI)	183 (87%)^a^	27.1 (24.5, 30.7)	26.9 (24.2, 30.5)	28.4 (25.3, 31.2)	0.2
Systolic blood pressure (mmHg)	183 (87%)^a^	126.0 (115.0, 138.0)	125.0 (114.3, 136.0)	130.0 (118.0, 140.0)	0.13
Diastolic blood pressure (mmHg)	183 (87%)^a^	80 (70, 85)	80 (70, 84)	81 (70, 87)	0.3
Pulse rate (beats per minute)	183 (87%)^a^	72 (67, 80)	72.0 (67, 80)	69 (64, 80)	0.15
Oxygen saturation (%)	183 (87%)^a^	97 (96, 98)	97 (96, 98)	96.5 (95, 98)	0.79
Lower limb edema	169 (80.4%)^b^	33 (19.5%)	19 (15.2%)	14 (31.8%)	0.03

Values are shown in absolute frequency and percentage or median and interquartile range.

NYHA, New York heart association; BMI, body-mass index.

^a^ 183 of 210 available; missing 27 (No MI: 19; MI: 8).

^b^ 169 of 210 available; missing 41 (No MI: 32; MI: 9).

* Pearson's Chi-squared test; Wilcoxon rank sum test.

Table 4 presents the results of psychiatric evaluations, assessed using the General Anxiety Disorder-7 (GAD-7), Patient Health Questionnaire-9 (PHQ-9), and Perceived Stress Scale-14 (PSS-14) questionnaires. Differences between the MI groups were observed only for PSS-14 scores >13 (*P* = 0.035).

**Table 4:** Psychiatric evaluation.

Variable	Overall, *N* = 195	No MI, *n* = 147	MI, *n* = 48	*P*-value*
PHQ-9 score	2.0 (0.0, 7.0)	2.0 (0.0, 7.0)	2.0 (0.0, 6.3)	0.6
PHQ-9 > 4	72 (36.9%)	57 (38.8%)	15 (31.3%)	0.4
GAD-7 score	1.0 (0.0, 5.0)	1.0 (0.0, 4.5)	3.0 (0.0, 6.3)	0.2
GAD-7 > 4	53 (27.2%)	37 (25.2%)	16 (33.3%)	0.4
PSS-14 score	5.0 (1.0, 12.0)	4.0 (1.0, 11.0)	6.0 (0.5, 16.0)	0.4
PSS-14 > 13	39 (20.1%)	24 (16.3%)	15 (31.9%)	0.035

Values are shown in absolute frequency and percentage or median and interquartile range.

GAD-7, general anxiety disorder-7; PHQ-9, patient health questionnaire-9; PSS-14, perceived stress scale-14.

* Fisher's exact test, Pearson's Chi-squared test; Wilcoxon rank sum test.

### 3.4 Clinical outcomes at follow-up

Medical follow-up after acute COVID-19 was conducted in 118 patients (75.1%) without MI and 47 patients (88.6%) with MI (*P* = 0.06). New-onset HTN occurred in 7 patients without MI, while no cases were observed in those with MI (*P* = 0.17). New-onset AF and heart failure (HF) were noted exclusively in patients with MI, affecting 3 (5.6%, *P* = 0.01) and 2 (3.7%, *P* = 0.06) patients, respectively.

VTE was identified in 6 patients (3.8%) without MI and 4 patients (7.5%) with MI (*P* = 0.27). Acute coronary syndrome (ACS) occurred in 3 patients (1.9%) without MI and 1 patient (1.9%) with MI (*P* > 0.9). Acute limb ischemia (ALI) was reported in 1 patient (0.6%) without MI and 1 patient (1.9%) with MI (*P* = 0.2). Stroke occurred in a single patient without MI (*P* > 0.9).

**Figure 2:**
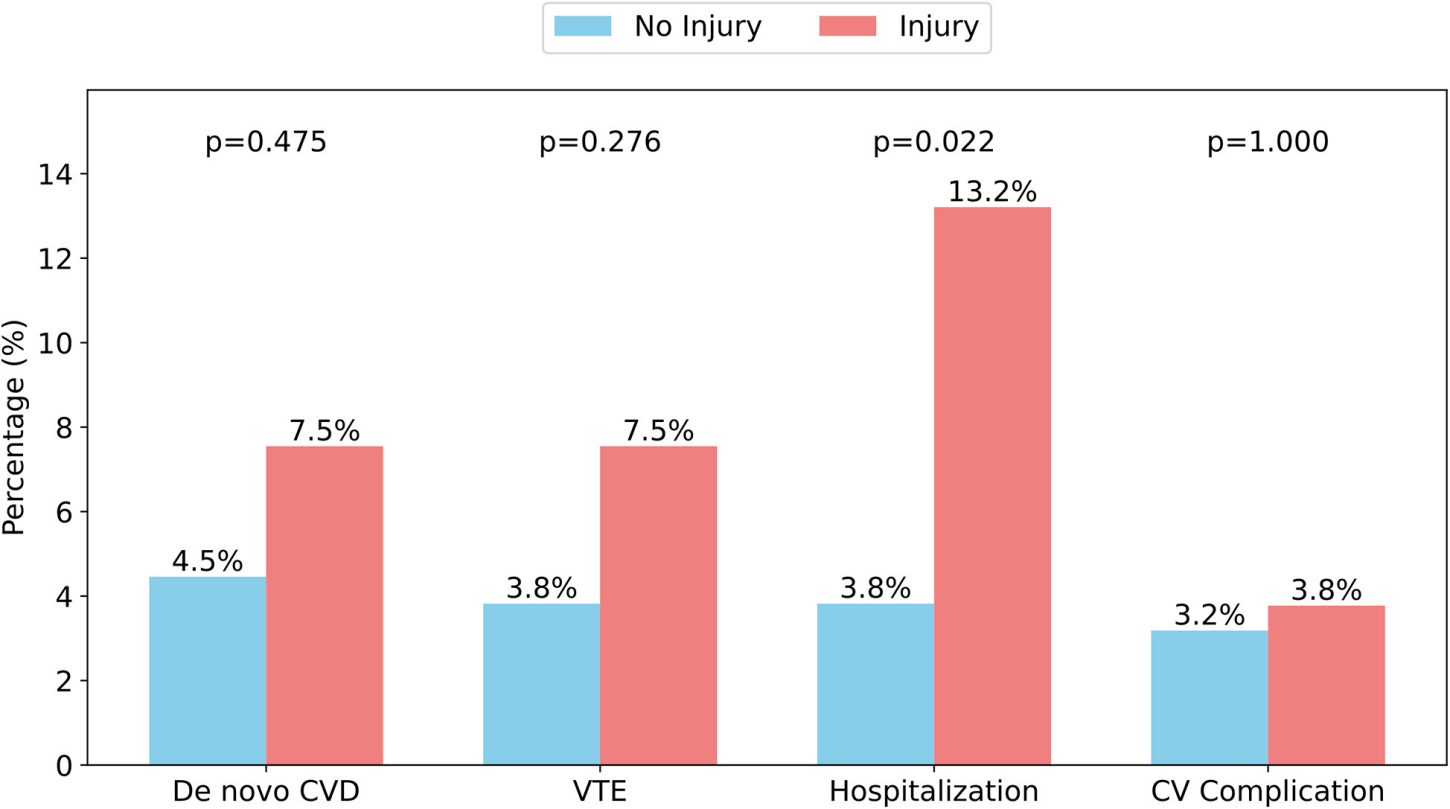
Incidence of clinical events stratified by MI. CVD, cardiovascular disease; VTE, venous thromboembolism; CV, cardiovascular. *De novo* CVD is the composite outcome of new-onset hypertension, new-onset atrial fibrillation, and new-onset heart failure. CV complication is the composite outcome of acute coronary syndrome, acute limb ischemia, and stroke. Hospitalization only included cardiopulmonary causes for in-patient admission.

Figure 2 illustrates the incidence of *de novo* cardiovascular disease (a composite of HTN, AF, and HF), VTE, cardiopulmonary hospitalizations, and cardiovascular complications (a composite of ALI, ACS, and stroke) between patients with and without MI.

In an effect-size logistic regression model, when adjusting for male sex (adjusted odds ratio, aOR 2.68, CI 95, 0.73–13.03, *P* = 0.16) and age at COVID-19 hospitalization (aOR 1.09, CI 95, 1.03–1.15, *P* = 0.001), a history of MI was associated with increased odds of cardiopulmonary hospitalization within two years of follow-up (aOR 3.67, CI 95, 1.07–13.07, *P* = 0.037).

### 3.5 Echocardiographic evaluation at follow-up

A full echocardiographic evaluation was completed in a subset of 147 (70%) patients, including 113 (71%) without MI and 34 (59%) with MI. No statistical differences were seen except in the number of patients with dilated atrium (defined as left atrial volume index of ≥29 ml/m^2^), which was 10.7% in the group without MI and 32.4% in the group with MI (*P* = 0.006). These findings are described in Table 5.

**Table 5:** Echocardiographic findings at follow-up stratified by MI.

Variable	Overall, *N* = 147	No MI, *n* = 113	MI, *N* = 34	*P*-value*
Left ventricular ejection fraction, %	65.0 (60.0, 65.0)	65.0 (60.0, 66.0)	63.0 (60.0, 65.0)	0.093
Left Ventricular End-Systolic Diameter, mm	29.0 (27.0, 32.0)	29.0 (27.0, 32.0)	30.0 (27.0, 33.0)	0.3
Left Ventricular End-Diastolic Diameter, mm	45.0 (41.0, 48.0)	45.0 (41.0, 48.0)	46.5 (42.3, 48.0)	0.3
Segmental wall motion abnormality	16 (10.9%)	9 (8.0%)	7 (20.6%)	0.79
Moderate to severe valvulopathy	8 (5.4%)	7 (6.2%)	1 (2.9%)	0.8
Dilated left atrium	23 (15.8%)	12 (10.7%)	11 (32.4%)	0.006

Values are shown in absolute frequency and percentage or median and interquartile range. The reduced sample size may introduce selection bias and reduce statistical power.

* Fisher's exact test, Pearson's Chi-squared test; Wilcoxon rank sum test.

## 4 Discussion

This is a multicenter prospective cohort study including patients who had severe COVID-19, 25% of whom had MI during hospitalization. We conducted comprehensive assessments of those who survived to a 2-year follow-up evaluation, encompassing demographic data, clinical variables, psychiatric assessments, and echocardiographic data. To the best of our knowledge, this is one of the longest follow-up periods for studies evaluating patients with previous MI in Latin America. The findings reveal that patients with prior MI had a higher prevalence of comorbidities, poorer functional status as determined by the NYHA classification, and greater odds of cardiopulmonary hospital admissions compared to those without MI. No differences were observed in the prevalence of cardiopulmonary symptoms or in the incidence of new-onset cardiovascular disease, VTE, or acute cardiovascular events.

In our study, patients with previous MI were older and had higher prevalences of comorbidities, an observation that has been reported multiple times in the literature (8, 10, 13, 19, 20, 21). These findings are expected as evidence demonstrates that patients with comorbidities, such as diabetes or coronary artery disease, are at increased risk of MI during acute COVID-19 (11). Patients with a history of MI showed a higher prevalence of a sedentary lifestyle compared to those without prior MI. One study showed that MI does not lead to reduced exercise capacity, although this study had a very small sample size (22). We believe that this might be explained by the increased multimorbidity in MI patients, but further evidence is needed to clarify MI's role in long-term functional status. Furthermore, patients with MI were more commonly admitted to the ICU, intubated, and treated with vasopressor or inotropic medications, which reinforces the increased risk of poor prognosis in this group (11). This is likely explained by the worse outcomes in COVID-19 patients with cardiovascular and metabolic comorbidities, as well as by direct cardiac damage in those with MI, and its complications, such as myocardial ischemia and myocarditis (11, 23).

COVID-19 survivors who had MI have a higher incidence of worse clinical outcomes in the long term. A Spanish study following up patients with MI for 6 months, found that they had higher readmission rates and mortality (11.6 vs. 1.16%, *P* = 0.013) (13). A large study including 4,695 participants, 1,168 (24.9%) who had MI during COVID-19 hospitalization, demonstrated a hazard ratio of 4.13 (95% CI 2.75–6.21) for mortality; the incidence of mortality in this group beyond 30 days after COVID-19 diagnosis was 6.8% compared to a 1.7% in those without MI (24). Another study with 701 patients, from which 75 had MI, demonstrated an increased probability of all-cause mortality and cardiovascular sequelae (e.g., arrhythmias and inflammatory heart diseases) in patients with MI at a median follow-up of 9 months (14). Another research including 377 COVID-19 survivors, found that those with MI had higher rates of readmissions and chronic sequelae of COVID-19 at 6 months (25).

Contrary to the findings reported in the mentioned studies, a matched cohort study comparing COVID-19 patients with MI to patients without COVID-19 or MI found no differences in major adverse cardiovascular events (MACE) or mortality at 12 months. However, an association with MACE was observed among patients with myocardial scars, as identified by baseline magnetic resonance imaging. Scars were more common in patients with elevated troponin levels during acute illness. These findings may suggest that the adverse events may be truly attributed to myocardial scar and not MI *per se* (26).

Our study found that patients with MI had higher risks of cardiopulmonary in-patient admissions during a two-year follow-up. However, we observed no significant differences in the incidence of *de novo* cardiovascular disease, VTE, or acute cardiovascular complications. Notably, unlike prior research, our study focused exclusively on patients who survived to the follow-up contact, which potentially introduced survivor bias that limited the detection of previously reported MI effects. Thus, our analysis could have underestimated the effect due to the exclusion of deceased patients, whose deaths could have been associated with these outcomes. Despite this underestimation, our data still indicate a slight trend toward a higher incidence of these outcomes in patients with MI, raising concerns about potential residual complications after 2 years. Based on the discussed studies and our findings, we emphasize the mixed evidence regarding the long-term outcomes of patients with MI, underscoring the need for further research to address this issue.

MI caused by COVID-19 can cause cardiac structural abnormalities acutely (27, 28), but the long-term structural consequences are still being investigated. A Spanish study evaluated 86 patients with a history of COVID-19 using echocardiography, including 43 patients with previous MI and 43 controls. The study found thicker ventricular walls in the MI group but no significant differences in other parameters (13). Another study from the United Kingdom used magnetic resonance imaging to evaluate this and found a higher prevalence of ventricular impairment and myocardial scarring in patients with a history of MI (26). In our study, we observed normal values for left ventricular ejection fraction and ventricular diameters. However, a significant proportion of patients with prior MI presented with segmental wall motion abnormalities and a dilated left atrium. This observation may be attributed to the increased prevalence of HF and AF observed in this group. Although a rigorous comparison with the group without injury is limited, the presence of numerous echocardiographic abnormalities in patients with MI highlights the need for further investigation into its role in the development of chronic structural heart abnormalities. This should be done while carefully accounting for the potential confounding effects of comorbidities.

Robust evidence demonstrates the substantial long-term psychological burden faced by patients after COVID-19, including high levels of anxiety and depression (29, 30). However, we found no data regarding the potential long-term psychiatric effects of patients with MI caused by COVID-19. This is particularly relevant due to the bidirectional correlation between cardiac disease and symptoms with mental health disturbances (31–33). We found one study that found that patients with previous MI had a higher prevalence of depression (16.7%) compared to those without MI (8.5%) (*P* = 0.043) at a median of 13 days follow-up (34). In our study, we found no substantial differences in PHQ-9, GAD-7, or PSS-14 overall scores between patients with and without MI. However, when applying a PSS-14 cutoff of >13 points, a notable difference was observed: approximately one-third of patients with MI reported moderate to high levels of perceived stress, compared to 15% of patients without MI. The specific cause of this association is difficult to determine due to the limited evidence available. One potential explanation is the known correlation between cardiovascular disease and perceived stress (35); however, further research is needed to clarify the potential link between MI and mental health.

Our findings add to the existing literature on risk assessment in COVID-19 survivors. Our findings add to the existing literature on risk assessment in COVID-19 survivors. Cardiovascular monitoring in COVID-19 patients is important, as even individuals who are not hospitalized may develop cardiovascular complications after recovering from the illness (36). Identifying patients at higher risk of cardiovascular sequelae, persistent symptoms, or psychiatric disturbances is crucial for effective prognostic stratification and the development of appropriate screening and follow-up strategies (14). More studies are required to elucidate the exact role of MI on long-term complications and their risk temporality. Further research is needed to clarify the precise role of MI in long-term complications and to better understand the timing and progression of associated risks.

## 5 Strengths and limitations

This study is one of the few published in Latin America that addresses long-term outcomes of patients with previous MI during acute COVID-19. It evaluates patients up to two years after the acute event and performs a comprehensive assessment including comorbidities, cardiovascular complications, physical examination, psychiatric evaluation, and cardiac imaging.

Potential selection bias due to missing troponin data was evident, as troponin testing appeared to be non-random and was associated with more severe clinical profiles. This selection bias means our findings primarily reflect the long-term outcomes of severe COVID-19 survivors who were deemed clinically relevant for troponin testing.

The comparison between the MI and no-MI groups occurs within this potentially higher-risk cohort, and excluding patients with potentially less severe initial illness (who did not have troponins measured) could influence the observed associations. For instance, the baseline characteristics of our “no MI” group might still represent a sicker population than the average hospitalized COVID-19 patient without measured troponins. This could lead to an underestimation of the relative difference in long-term outcomes between MI and truly “lower-risk” no-MI patients, or conversely, it might influence the types of long-term outcomes observed. Therefore, while our study provides valuable insights into the long-term sequelae in survivors of severe COVID-19 with available troponin data, the results should be interpreted with caution, acknowledging that they may not be fully generalizable to the entire population of hospitalized COVID-19 patients due to this potential selection bias.

A further limitation is the lack of standardization in the timing of troponin measurements. While troponin levels were obtained at admission, discharge, or both, this variability limits the ability to assess troponin dynamics or establish temporal patterns of MI. Some transient or late-onset elevations may have been missed, and the interpretation of MI may differ depending on the timing of measurement. Nonetheless, for consistency, MI was operationally defined as any troponin elevation during hospitalization, regardless of timing.

Another caveat is the possible survivor bias. Since follow-ups were conducted at the 2-year mark, patients who did not survive or were unreachable could not be included in the analysis. Therefore, these findings should be interpreted cautiously, as they primarily apply to patients who survived for at least two years following acute COVID-19. Another limitation is the definition of severe COVID-19, which was used according to the original CARDIO COVID 19–20 study and not other official classifications such as the one by the World Health Organization. In addition, many institutions did not participate in this follow-up study, and a substantial number of patients were lost to follow-up, resulting in a relatively small sample. This impacted the number of outcome events in our study, limiting our ability to adjust for some confounding factors.

Missing data likely resulted from various factors, such as the limited number of in-person assessments (only 183, 87% of the total sample) and patients declining imaging. Another limitation is the potential for recall bias in symptom reporting, especially in elderly participants. To minimize this, participants were specifically asked about symptoms experienced during the three months preceding the evaluation. However, this may still affect the reliability of the reported symptom frequencies in our cohort.

Lastly, most participants were from Colombia, so the findings might not represent other Latin American countries equally.

## 6 Conclusion

Patients with prior MI during COVID-19 had a higher prevalence of comorbidities, poorer functional status, and increased odds of cardiopulmonary hospitalizations over a two-year follow-up evaluation compared to those without MI. There were no substantial differences in the prevalence of cardiopulmonary symptoms, abnormal psychiatric evaluations, or the incidence of new-onset cardiovascular disease, VTE, and acute cardiovascular complications. Although prior studies suggest an association between MI and worse long-term outcomes, such as increased mortality and structural cardiac abnormalities, the evidence remains inconsistent. These findings emphasize the need for ongoing research to clarify how MI contributes to chronic disease and worsened long-term outcomes.

## Data Availability

The data supporting the conclusions of this manuscript will be made available by the corresponding author upon reasonable request.
