# Supplementary material for: Two-year follow-up of patients with myocardial injury during acute COVID-19: insights from the CARDIO COVID 20–21 registry
Source: Front Cardiovasc Med. 2025 Jun 11;12:1584732. doi: 10.3389/fcvm.2025.1584732 (PMC12187653; doi:10.3389/fcvm.2025.1584732)
Supplement: Supplementary file 1 [file Table1.docx]

# **SUPPLEMENTARY MATERIAL**

**Table 1. Distribution of troponin measurement timing and positivity status (n = 210)**

| **Measurement scenario** | **n (%)** |
| --- | --- |
| **Only admission measurement** |  |
| Positive | 27 (12.9%) |
| Negative | 109 (51.9%) |
| **Admission and discharge measurements** |  |
| Both positive | 18 (8.6%) |
| Positive at admission, negative at discharge | 7 (3.3%) |
| Negative at admission, positive at discharge | 0 (0.0%) |
| Both negative | 45 (21.4%) |
| **Only discharge measurement** |  |
| Positive | 1 (0.5%) |
| Negative | 3 (1.4%) |

Values are shown in absolute frequency and percentage

**Table 2. Baseline characteristics during acute COVID-19 of patients with and without troponin measurement**

| **Variable** | **Troponin measured, n = 2,134** | **Troponin not measured, n = 1,126** | **P-value*** |
| --- | --- | --- | --- |
| **Demographics** |  |  |  |
| Age (years) | 61 (49, 71) | 61 (48, 71) | 0.4 |
| Sex (Male) | 1,372 (64%) | 688 (61%) | 0.072 |
| **Comorbidities** |  |  |  |
| Arterial hypertension | 1,045 (49%) | 551 (49%) | >0.9 |
| Dyslipidemia | 316 (15%) | 135 (12%) | 0.027 |
| Diabetes mellitus | 594 (28%) | 275 (24%) | 0.036 |
| Chronic kidney disease | 158 (7.4%) | 112 (9.9%) | 0.012 |
| Coronary artery disease | 186 (8.7%) | 58 (5.2%) | <0.001 |
| Heart failure | 127 (6.0%) | 55 (4.9%) | 0.2 |
| Atrial fibrillation | 74 (3.5%) | 41 (3.6%) | 0.8 |
| Stroke | 62 (2.9%) | 40 (3.6%) | 0.3 |
| **Baseline medications** |  |  |  |
| ARB-II | 550 (26%) | 255 (23%) | 0.049 |
| Beta blockers | 117 (10%) | 315 (15%) | <0.001 |
| ACEi | 230 (11%) | 128 (11%) | 0.6 |
| Antiplatelet | 246 (12%) | 105 (9.3%) | 0.054 |
| Statin | 279 (13%) | 119 (11%) | 0.038 |
| Anticoagulant | 57 (5.1%) | 94 (4.4%) | 0.4 |
| ARA | 64 (3.0%) | 37 (3.3%) | 0.7 |
| SGLT-2i | 21 (1.0%) | 7 (0.6%) | 0.3 |
| **In-hospital management** |  |  |  |
| Corticosteroids | 1,466 (69%) | 727 (65%) | 0.017 |
| Anticoagulation | 786 (37%) | 464 (41%) | 0.015 |
| IMV | 842 (39%) | 269 (24%) | <0.001 |
| **In-hospital outcomes** |  |  |  |
| ICU admission | 1,292 (61%) | 447 (40%) | <0.001 |
| Death | 540 (25.3%) | 287 (25.5%) | >0.9 |

Abbreviations: ARBII, Angiotensin II receptor blocker; ACEi, Angiotensin-converting enzyme inhibitor; ARA, Aldosterone receptor antagonist; ICU, Intensive care unit; IMV, Invasive mechanical ventilation; SGLT-2i, Sodium-glucose co-transporter-2 inhibitor.

Values are shown in absolute frequency and percentage, or median and interquartile range.

*Fisher's exact test, Pearson’s Chi-squared test; Wilcoxon rank sum test

**Table 3. Baseline characteristics during acute COVID-19 of included and excluded participants from participating institutions of CARDIO COVID 20-21**

| **Variable** | **Excluded, n = 243** | **Included, n = 210** | **P-value*** |
| --- | --- | --- | --- |
| Myocardial injury | 76 (31%) | 53 (25%) | 0.175 |
| **Demographics** |  |  |  |
| Age (years) | 57 (46, 69) | 58 (49, 69) | 0.3 |
| Sex (Male) | 141 (58%) | 130 (62%) | 0.442 |
| **Comorbidities** |  |  |  |
| Arterial hypertension | 113 (47%) | 97 (46%) | >0.9 |
| Dyslipidemia | 20 (8.2%) | 25 (12%) | 0.21 |
| Diabetes mellitus | 55 (23%) | 47 (22%) | >0.9 |
| Chronic kidney disease | 19 (7.8%) | 14 (6.7%) | >0.9 |
| Coronary artery disease | 14 (5.8%) | 12 (5.7%) | >0.9 |
| Heart failure | 6 (2.5%) | 8 (3.8%) | 0.24 |
| Atrial fibrillation | 6 (2.5%) | 8 (3.8%) | 0.24 |
| Stroke | 8 (3.3%) | 4 (1.9%) | 0.362 |
| **Baseline medications** |  |  |  |
| ARB-II | 76 (31%) | 57 (27%) | 0.451 |
| Beta blockers | 32 (13%) | 27 (13%) | >0.9 |
| ACEi | 16 (6.6%) | 12 (5.7%) | >0.9 |
| Antiplatelet | 26 (11%) | 17 (8.1%) | 0.435 |
| Statin | 24 (9.9%) | 30 (14%) | 0.2 |
| Anticoagulant | 7 (2.9%) | 6 (2.9%) | >0.9 |
| ARA | 3 (1.2%) | 9 (4.3%) | 0.028 |
| SGLT-2i | 3 (1.2%) | 2 (1.0%) | >0.9 |
| **In-hospital management** |  |  |  |
| Corticosteroids | 190 (81.3%) | 177 (83.8%) | 0.72 |
| Anticoagulation | 69 (28%) | 64 (30%) | 0.682 |
| IMV | 68 (28%) | 59 (28%) | 0.74 |
| **In-hospital outcomes** |  |  |  |
| ICU admission | 151 (62%) | 122 (58%) | 0.544 |

Abbreviations: ARBII, Angiotensin II receptor blocker; ACEi, Angiotensin-converting enzyme inhibitor; ARA, Aldosterone receptor antagonist; ICU, Intensive care unit; IMV, Invasive mechanical ventilation; SGLT-2i, Sodium-glucose co-transporter-2 inhibitor.

Values are shown in absolute frequency and percentage, or median and interquartile range.

*Fisher's exact test, Pearson’s Chi-squared test; Wilcoxon rank sum test
